# Does the chronic care model meet the emerging needs of people living with multimorbidity? A systematic review and thematic synthesis

**DOI:** 10.1371/journal.pone.0190852

**Published:** 2018-02-08

**Authors:** Kasey R. Boehmer, Abd Moain Abu Dabrh, Michael R. Gionfriddo, Patricia Erwin, Victor M. Montori

**Affiliations:** 1 Knowledge and Evaluation Research (KER) Unit, Mayo Clinic, Rochester, Minnesota, United States of America; 2 Department of Family Medicine, Mayo Clinic Florida, Jacksonville, Florida, United States of America; 3 Center for Pharmacy Innovation and Outcomes, Geisinger, Forty Fort, Pennsylvania, United States of America; 4 Mayo Medical Libraries, Mayo Clinic, Rochester, Minnesota, United States of America; Universita degli Studi di Firenze, ITALY

## Abstract

**Background:**

The Chronic Care Model (CCM) emerged in the 1990s as an approach to re-organize primary care and implement critical elements that enable it to proactively attend to patients with chronic conditions. The chronic care landscape has evolved further, as most patients now present with multiple chronic conditions and increasing psychosocial complexity. These patients face accumulating and overwhelming complexity resulting from the sum of uncoordinated responses to each of their problems. Minimally Disruptive Medicine (MDM) was proposed to respond to this challenge, aiming at improving outcomes that matter to patients with the smallest burden of treatment. We sought to critically appraise the extent to which MDM constructs (e.g., reducing patient work, improving patients’ capacity) have been adopted within CCM implementations.

**Methods:**

We conducted a systematic review and qualitative thematic synthesis of reports of CCM implementations published from 2011–2016.

**Results:**

CCM implementations were mostly aligned with the healthcare system’s goals, condition-specific, and targeted disease-specific outcomes or healthcare utilization. No CCM implementation addressed patient work. Few reduced treatment workload without adding additional tasks. Implementations supported patient capacity by offering information, but rarely offered practical resources (e.g., financial assistance, transportation), helped patients reframe their biography with chronic illness, or assisted them in engaging with a supportive social network. Few implementations aimed at improving functional status or quality of life, and only one-third of studies were targeted for patients of low socioeconomic status.

**Conclusion:**

MDM provides a lens to operationalize how to care for patients with multiple chronic conditions, but its constructs remain mostly absent from how implementations of the CCM are currently reported. Improvements to the primary care of patients with multimorbidity may benefit from the application of MDM, and the current CCM implementations that do apply MDM constructs should be considered exemplars for future implementation work.

## Introduction

In the 1990s, Wagner and colleagues developed the evidence-based Chronic Care Model (CCM). The CCM had significant advantages over the primarily acute-care model of primary care at the time. Namely, it responded to the need for the healthcare system to change structurally how it addressed the needs of patients with chronic illness.[1] The CCM oriented primary care’s shift to proactive management of chronic conditions.[2] Two decades later, the CCM has been packaged into toolkits[3, 4] and widely adopted. In that time, though, the landscape of chronic care has further changed.

In 2009, a new problem in the care of patients with multimorbidity, i.e., the coexistence and interaction of *multiple chronic conditions* (MCC); a growing public health problem that affects 3 in 4 Americans 65 and older,[5, 6] was recognized. Some patients were unable to complete all tasks assigned to them because of the way care was organized and delivered. Usual care was transferring to these patients more work than what their capacity could enact. A solution, Minimally Disruptive Medicine (MDM),[7] proposed that health care should account for patient work, should work to make it fit in the context and work of living, and seek to achieve patient goals while minimizing the burden of treatment. In the past eight years, this model has begun to gain traction.[8] Supported by a conceptual and theoretical foundation,[9–15] MDM is responsive to the accumulation of chronic conditions that is increasingly prevalent. Its main contribution is to orient healthcare to rightsize the work delegated to the patient and support the patient’s capacity to enact it.[7]

MDM builds on the CCM to address two of its weaknesses. First, the CCM describes what elements should be implemented to support patients with chronic conditions, but not how these implementations should handle multimorbidity. Conceivably, the CCM could simply be applied to handle multiple individual conditions. However, there is a growing body of evidence that shows that disease and treatment interactions, and interactions between the biomedical and the socio-personal context of each patient, make it unwise to care for each condition separately (i.e., as when each one is handled by uncoordinated specialists) and call for whole-person primary care for patients with multimorbidity.[16–21] Such patients and their caregivers may become overwhelmed by chronic care that ignores the accumulation of tasks, all recommended in the care of each condition.[10, 11, 22–24]

Second, in its original conception, components of the CCM were assembled based on favorable experience with each component independently, rather than to respond to the tenets of a conceptual or theoretical framework. The sum of the components may not preserve their advantages or achieve synergies. MDM’s theoretical and conceptual frameworks may guide the implementation of CCM’s elements to patients with MCC. An additional advantage is that interventions that seek to apply theoretical and conceptual foundations may be more effective.[25]

MDM has a conceptual model, the Cumulative Complexity Model (CuCoM), and two middle-range theories relevant to this manuscript: the Normalization Process Theory (NPT) and the Theory of Patient Capacity (known by its pneumonic, BREWS). CuCoM describes the cumulative work of implementing healthcare and self-care tasks for patients with multimorbidity, and how without consideration of patients’ other conditions and of their life situation, this work can overwhelm the capacity (i.e. abilities and resources)[15] of patients and their caregivers to enact treatment plans.[7, 10, 11, 14, 26] Practically, this translates into a choice between enacting and adhering to treatment or engaging in life duties, roles and activities; in choosing the latter, as most patients do,[7] patients may delay or cancel healthcare tasks, becoming labeled as “noncompliant”.

NPT offers a more in-depth explanation of the nature of patient work. This includes making sense of the work required (e.g., reading pamphlets, thinking through how to adhere to the treatment regimen), enrolling others to help and planning the work, conducting the work (e.g. attending the appointments, successfully adhering to treatment), and appraising, continuously, whether the work is worth the effort.[10–13] For patients with chronic conditions, many of which are asymptomatic, the appraisal is complicated by the absence of or delayed feedback from the condition. Patient work was described before the CCM’s genesis[27] and has been described in later qualitative research specifically relating to multimorbidity,[28] but its importance was not acknowledged in the original CCM[1] or in later versions of the model.[29] Finally, the Theory of Patient Capacity puts forth that patients’ capacity to take on the self-care tasks are resultant of their interactions with their **B**iography and their ability to incorporate their illness and its care into that biography, **R**esources, **E**nvironment, experiential learning from the **W**ork of being a patient, and **S**ocial network (BREWS).

### The present review

To date, no review of the literature addresses the extent to which the elements of MDM, namely those constructs described in the CuCoM, NPT, and BREWS, have guided the implementation of the CCM. Thus, we sought to critically appraise reports of the implementations of the CCM to address this knowledge gap.

## Methods

To explore the extent to which MDM constructs are present in the reporting of current CCM implementations, we conducted a systematic review and thematic synthesis following the ENTREQ reporting guidelines (S1 Table).[30]

### Study eligibility

We included English-language studies published within the last 5 years (July 2011- July 2016) describing implementations of the CCM using any study design. We chose the past 5 years to capture contemporary practice rather than historical trends, and to give time for implementers to consider MDM (its first description was published in 2009). Eligible studies had to state that their intervention was based on the CCM, and to describe implementing at least one of the five components of the original CCM: 1) the use of evidence-based, planned care and protocols; 2) practice redesign to meet the needs of patients with chronic conditions, in terms of additional time and close follow-up; 3) patient self-management and behavioral change support; 4) ready access to clinical expertise; and/or 5) supportive information systems.^1^ We excluded protocol papers for planned studies; however, if an included study had an available protocol (as an appendix to the study or as a standalone publication), we reviewed the methods reported in all these sources.

### Search strategy

An expert reference librarian (P.E.) created and conducted the initial search from July 2011 to July 2016 using the Ovid MEDLINE and Scopus databases (See S1 Text for full search strategies). We also reviewed the references of included studies and of systematic reviews for potentially eligible studies.

### Selection of studies

Prior to beginning screening for study eligibility, two reviewers (K.B. and M.G.) were trained regarding the purpose of the review and eligibility criteria. They conducted abstract and full text screening in duplicate, with disagreements at abstract screening included in full-text screening. Full-text screening disagreements were resolved by discussion and consensus.

### Data extraction and quality assessment

We extracted in duplicate pertinent study characteristics, CCM components targeted in the intervention and any additional theoretical frameworks used, using a systematic review software, Distiller SR (EvidencePartners, Ottawa, Canada). Quality was assessed in duplicate using the “Template for Intervention Description and Replication (TIDieR) checklist.[31] This checklist is designed to assess the completeness of intervention descriptions, the clarity of the proposed mechanisms for change, and how well the intervention was implemented.[31] All disagreements were resolved by consensus.

### Data analysis

Articles were imported into qualitative data analysis software (NVivo^®^ qualitative data analysis Software; QSR International Pty Ltd. Version 10, 2014). In order to synthesize the overarching themes of current CCM implementations, we conducted an inductive thematic synthesis.[32] Traditionally, this method has been applied to synthesize textual findings during systematic reviews of qualitative studies without *a priori* expectations. Because we aimed to synthesize textual information slightly different in nature, about how interventions were enacted, but without preconceived *a priori* expectations, we selected this method. Ultimately, thematic synthesis is “the process of taking concepts from one study and recognising the same concepts in another study, though they may not be expressed using identical words,”[32] which facilitates a summary of what is happening across many interventions. Using previously described thematic synthesis methods,[32] two coders (K.B., M.A.) first coded five studies line-by-line to create the initial list of codes. During this process, each segment of text is described by a “code” (e.g., adherence, coaching, patient skill building). The coders then met to discuss and refine this list, deleting duplicate codes, combining similar ideas into individual codes, and resolving coding discrepancies. They then coded three additional studies in duplicate using the refined coding list, and added additional codes that emerged during the process. They again met to discuss this process and finalize the coding scheme. Reviewers completed coding the remainder of studies individually, and met weekly to discuss any newly emerging codes and questions. Once the coding was completed, K.B. synthesized codes into overarching themes.

K.B. then compared intervention characteristics and themes that emerged from analysis with the tenets of MDM, using the CuCoM, NPT and BREWS.[9, 12, 13, 15] Using the CuCoM,[15] each study was categorized as adding patient work (+), subtracting patient work (-), neutral to patient work (N), meaning it both added and subtracted patient work, or as having an unclear effect. Using NPT,[12, 13] we identified if the intervention assisted patients with sense-making work (S), enrolling others to help, and planning the work (E), enacting the patient work (W), or appraising the work (A). Using BREWS,[9] we identified if each intervention supported patients’ capacity by helping them reframe their biography with chronic illness (B), provided or assisted in accessing resources (R), improved the environment in which patients received care (E), promoted experiential success in managing the work of healthcare and life (W), or supported the patients interaction with their social network (S).

## Results

### Identification of studies

**Fig 1:**
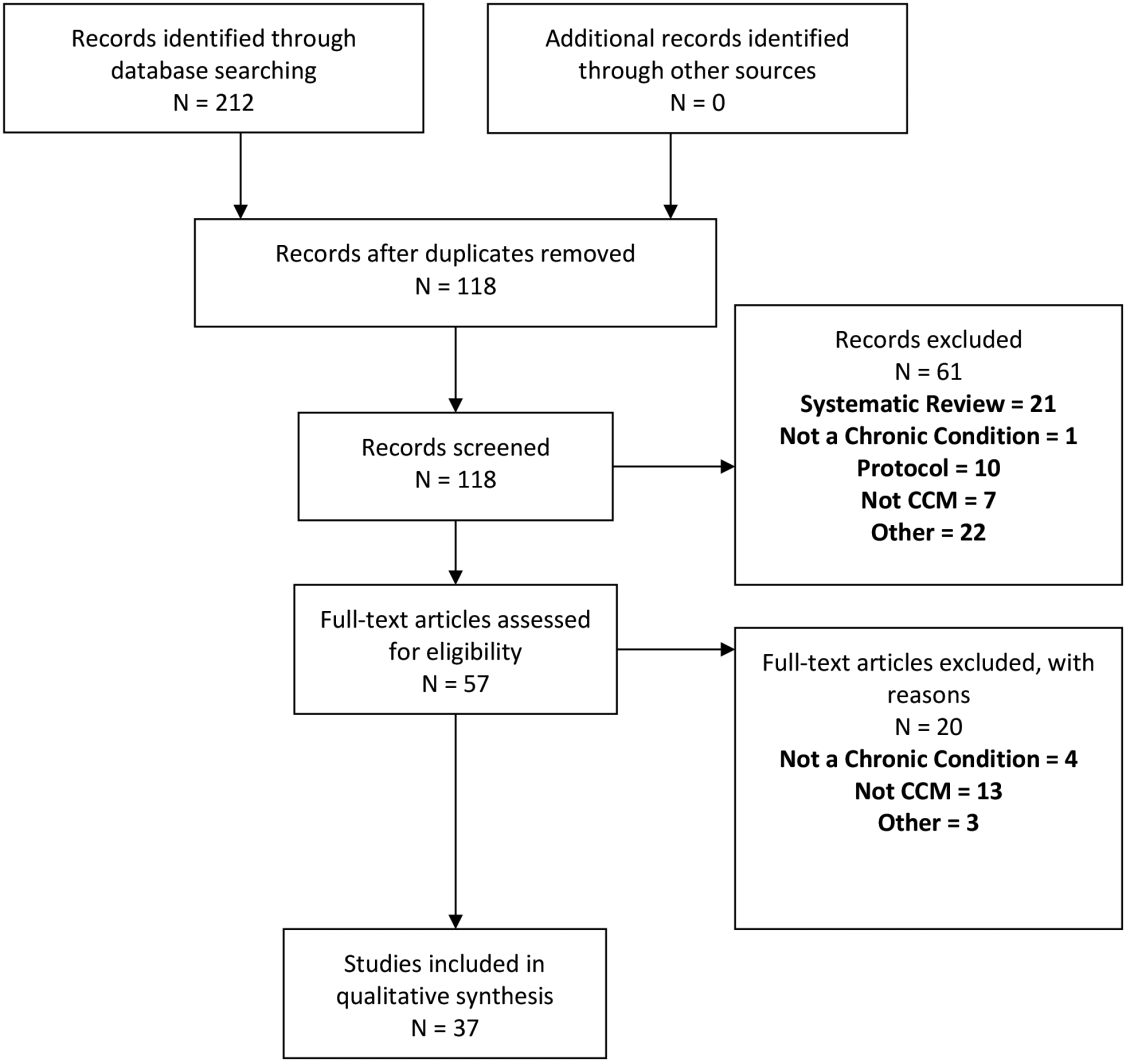
PRISMA study eligibility and inclusion process.

The initial search yielded 118 potentially eligible articles, of which 37 reports of 29 studies were included with sufficient chance-adjusted inter-rater reliability (κ = 0.78; Fig 1)

### Summary of included studies

Table 1 describes the included studies. Most articles described quantitative analyses (n = 24) of implementations focused on a single condition (e.g., chronic obstructive pulmonary disease, asthma, chronic kidney disease), most commonly type 2 diabetes, and implemented patient self-management support and practice redesign. Very few addressed patients with comorbidities (n = 3) or were condition agnostic (n = 4).

**Table 1:** Study characteristics.

Author	Year	Type	Conditions	EBP	Redesign	SMS	Expertise	SIS	Duration	Framework	Conflicts
Austin	2013	Quant	Type II Diabetes			**X**			4 weeks; support group for 12 months	None	No
Bissonnette	2013	Quant	Chronic Kidney Disease	**X**	**X**	**X**	**X**		3.5 years	None	No
Bojadzievski	2012	Quant	Type II Diabetes/Hyperlipidemia					**X**	Unclear	None	No
Britto	2014	Quant	Asthma	**X**	**X**	**X**		**X**	4 years	None	No
Collinsworth	2014	Qual	Type II Diabetes		**X**	**X**			18 months	none	No
Comín-Colet	2014	Quant	Heart Failure	**X**	**X**	**X**	**X**	**X**	6 years	None	no
Crabtree	2014	Mixed	Hypertension		**X**	**X**			unclear	Model for Improvement	No
Cramm	2014	Mixed	Type II Diabetes/Heart Failure/Comorbidities/COPD/Cardiovascular Disease	**X**	**X**	**X**		**X**	1 year	none	No
Cramm	2014	Quant	Type II Diabetes/Depression/Heart Failure/Comorbidities/COPD/Cardiovascular Disease/Stroke/Eating Disorders	**X**	**X**	**X**		**X**	2 years	none	No
Cramm	2012	Quant	Type II Diabetes/Depression/Heart Failure/Comorbidities/COPD/Cardiovascular Disease/Stroke/Eating Disorders/Psychotic Disorders	**X**	**X**	**X**	**X**	**X**	1 year	None	No
Dickinson	2014	Quant	Type II Diabetes	**X**	**X**	**X**	**X**	**X**	6–18 months	Complexity Theory; Model for Improvement	No
Dickinson	2014	Quant	Type II Diabetes		**X**			**X**	12–18 months	None	No
Farley	2014	Quant	Tuberculosis	**X**	**X**	**X**	**X**	**X**	6 months	PRECEED-PROCEED	No
Goldwater	2014	Qual	Type II Diabetes/Hypertension/Hyperlipidemia/Tuberculosis	**X**				**X**	Unclear	None	No
Halladay	2014	Quant	Type II Diabetes	**X**		**X**		**X**	13+ months	none	No
Hariharan	2014	Quant	Type II Diabetes	**X**	**X**	**X**	**X**	**X**	3 years	none	No
Heinelt	2015	Mixed	Not Targeted		**X**		**X**	**X**	unclear	none	No
Holm	2014	Qual	Depression		**X**	**X**			12 months	none	No
Holtrop	2015	Mixed	Type II Diabetes		**X**	**X**		**X**	9 months	Macrocognition Framework	No
Ku	2015	Mixed	Type II Diabetes	**X**	**X**	**X**		**X**	28 months	none	No
Ku	2014	Quant	Type II Diabetes	**X**	**X**	**X**			22 months	none	No
Langwell	2014	Mixed	Type II Diabetes			**X**			4 years	none	No
Mackey	2012	Quant	Type II Diabetes	**X**	**X**	**X**	**X**	**X**	Unclear	None	No
Martin	2016	Quant	Not Targeted			**X**			Unclear	Bandura’s Social Cognitive Theory	No
Massoud	2015	Quant	HIV		**X**	**X**		**X**	Unclear	Systems theory; Model for Improvement	No
McGough	2016	Quant	Depression/Anxiety	**X**	**X**	**X**	**X**	**X**	44 months	none	No
Noel	2014	Quant	Type II Diabetes	**X**	**X**	**X**		**X**	12 months	None	No
Parchman	2013	Quant	Type II Diabetes	**X**	**X**	**X**	**X**	**X**	1 year	None	No
Philis-Tsimikas	2014	Qual	Type II Diabetes		**X**	**X**		**X**	Varying	None	No
Pilleron	2014	Quant	Type II Diabetes	**X**	**X**	**X**	**X**	**X**	3 years	none	No
Roland	2012	Quant	COPD or Not Targeted		**X**	**X**		**X**	6 months	None	No
Sack	2012	Quant	Inflammatory Bowel Disease	**X**	**X**	**X**	**X**	**X**	5 months	None	No
Schauer	2013	Qual	Not Targeted	**X**	**X**	**X**	**X**	**X**	Unclear	None	No
Smidth	2013	Qual	COPD	**X**	**X**	**X**		**X**	25 months	Medical Research Council’s framework	No
Smidth	2013	Quant	COPD	**X**	**X**	**X**		**X**	25 months	None	No
Tu	2013	Quant	HIV	**X**	**X**	**X**	**X**	**X**	3 years	None	Yes
Van Durme	2015	Mixed	Not Targeted	**X**	**X**	**X**	**X**	**X**	15 days—36 months; mean 6 months	Complexity Theory	No

### Protection from bias and reporting of methods

With few exceptions, most studies used methods warranting trustworthy results (S2 Table). However, intervention fidelity assessments were rare. For example, studies that included patient self-management support sessions did not assess the extent to which the curriculum was covered or patients attended the sessions. Several studies described poorly how the intervention was delivered, i.e., in-person or online, or how to access the materials used.

### Major themes

The inductive thematic synthesis highlighted four high-level themes: intervention aims, practice assessment mechanisms, intervention alignment with different healthcare stakeholders, and the ways in which practices assisted patients with self-management. Each of these could be broken down into subthemes (Table 2).

**Table 2:** Themes of CCM implementation with examples.

Theme	Sub-Themes	Representative Quotes
**Aims**	Adherence to treatment; implemeting behavioral changes; improving disease-specific outcomes; reducing healthcare utilization; improving functional status or overall well-being; quality of life	*“The RNs provided outreach for continued motivation and adherence and providers integrated the information from each patient’s HBPM diary into their treatment strategy.”* Crabtree, 2014 *“The health coach describes this: “I help keep them compliant… make sure they’re seeing their doctor on time, they’re keeping their appointments, they, they get a wellness check and they get a physical each year… to make sure they’re doing that. If you are diabetic, I’m making sure that you are doing what you’re supposed to—getting your A1Cs, checking blood sugar on time, taking any meds.””* Shauer, 2013
**Alignment**	Healthcare system; community; patients; clinicians	*“Defy Diabetes! created a unique collaborative partnership between Seton Health, CDEs, faith community nurses and churches, and a number of other key partners such as other medical centers, the local ADA chapter, several colleges and universities, and Cornell Cooperative Extension to impact diabetes in the community.”* Austin, 2013
**Assessment**	EHR; patient registries; quality ratings, patient satisfaction	*“The presence and use of an electronic patient record and a registry, including a list of beneficiaries of the projects and reminders to providers to plan care were important facilitators of the process.”* Van Durme, 2015
**Assisting**	Care coordination; collaboration with other clinical teams and community agencies; team-based care; financial assistance; patient education; overcoming patient barriers; changing the flow and feel of the care environment; coping support	*“[Diabetes self-management education] DSME sessions focused on: information on diabetes and diabetes medications, adoption of self-care behaviour, gaining control over the condition through problem solving skills and goal setting.”* Ku, 2014 *“Scheduled phone follow-up for any patient with symptoms at routine clinic visits and post hospital discharge to ensure resolution (pre-empting any deterioration whilst awaiting next routine visit).”* Sack, 2011 *“The social worker also assessed the patient during the clinic visit reviewing advanced care directives, financial, or social support issues identified during the interaction. The social worker assessed the patient’s overall coping response to his or her chronic kidney disease and inquired about any major life changes (e.g., death, job loss, etc.).”* Woodend, 2013

The primary aims of CCM interventions were: understanding characteristics of successful or unsuccessful implementation, improved adherence to therapy, behavioral changes, decreased healthcare utilization, improvement in disease-specific outcomes, and in a few cases, patient-reported outcomes such as quality of life, functional status, wellness, or coping. Most studies aligned their aims with the healthcare system administration as the primary stakeholder. Only the few studies that involved patients, the community, or practicing clinicians as stakeholders in the development and implementation of CCM interventions aligned their aims with them.

The primary method for assessing the success of the intervention included collection of data in the electronic medical record or patient registries. A small number of studies used quality improvement methods, such as rating systems. The rest used the number of patients receiving or referred to specific services, patient satisfaction, and the score on the Assessment of Chronic Illness Care, a measure of organizational alignment with the CCM, reported by clinicians and health professionals at an institution.

### Current CCM implementation versus the principles of MDM

Constructs of MDM that were described in the CCM implementations are reported in Table 3, using the CuCom, NPT, and BREWS.[9, 12, 13, 15]

**Table 3:** Study-by-Study look at the inclusion of MDM constructs and study outcome reporting.

Author	Workload	NPT (normalizing the workload)	Capacity	Outcomes Reported (Y/N)	Outcome Focus	Outcomes
**Austin**	**+**	**S**EWA	B**REW**S	**Y**	**Both**	**N**
**Bissonnette**	**N**	**SE**WA	**BREW**S	**Y**	**System**	**+**
**Bojadzievski**	**+**	SEWA	BREWS	**N**	**N/A**	**N/A**
**Britto**	**N**	**SEWA**	**BREWS**	**Y**	**Both**	**+**
**Collinsworth**	**N**	**SEW**A	B**REWS**	**N**	**N/A**	**N/A**
**Comín-Colet**	**N**	**SEW**A	B**REWS**	**Y**	**System**	**+**
**Crabtree**	**+**	**S**EWA	B**R**E**W**S	**Y**	**System**	**N**
**Cramm**	**+**	**S**EW**A**	B**REW**S	**Y**	**Patient**	**N**
**Cramm**	**+**	**S**EW**A**	B**REW**S	**Y**	**System**	**+**
**Cramm**	**+**	**S**EW**A**	B**REW**S	**Y**	**System**	**N**
**Dickinson**	**+**	SEWA	BREWS	**N**	**N/A**	**N/A**
**Dickinson**	**Unclear**	SEWA	BREWS	**N**	**N/A**	**N/A**
**Farley**	**Unclear**	SEWA	BR**E**WS	**Y**	**System**	**+**
**Goldwater**	**+**	**S**EWA	BREWS	**N**	**N/A**	**N/A**
**Halladay**	**Unclear**	**S**EWA	BREWS	**Y**	**System**	**N**
**Hariharan**	**+**	**S**EWA	B**RE**WS	**Y**	**System**	**+**
**Heinelt**	-	**SEW**A	B**RE**WS	**N**	**N/A**	**N/A**
**Holm**	**Unclear**	SEWA	BREWS	**N**	**N/A**	**N/A**
**Holtrop**	**unclear**	**S**EWA	B**RE**WS	**Y**	**System**	**N**
**Ku**	**+**	**S**EWA	B**R**EWS	**Y**	**System**	**+**
**Ku**	**+**	**SE**WA	B**R**E**W**S	**Y**	**Both**	**N**
**Langwell**	**+**	**S**EWA	B**RE**WS	**N**	**N/A**	**N/A**
**Mackey**	**+**	**S**EWA	B**R**EW**S**	**N**	**N/A**	**N/A**
**Martin**	**+**	**S**E**W**A	B**R**E**W**S	**Y**	**Patient**	**+**
**Massoud**	-	S**EW**A	B**REW**S	**Y**	**System**	**+**
**McGough**	**N**	**SE**WA	B**REW**S	**Y**	**System**	**+**
**Noel**	**+**	**S**EWA	B**R**EW**S**	**N**	**N/A**	**N/A**
**Parchman**	**+**	**S**EWA	B**R**EW**S**	**Y**	**System**	**+**
**Philis-Tsimikas**	**N**	**SEW**A	**BREWS**	**N**	**N/A**	**N/A**
**Pilleron**	**+**	**SE**WA	B**REW**S	**Y**	**System**	**N**
**Roland**	-	S**EW**A	B**REW**S	**Y**	**Both**	**N**
**Sack**	-	**S**E**WA**	B**RE**W**S**	**Y**	**System**	**+**
**Schauer**	**+**	**S**EWA	B**REW**S	**N**	**N/A**	**N/A**
**Smidth**	**N**	**SEWA**	**BREWS**	**N**	**N/A**	**N/A**
**Smidth**	**N**	**SEWA**	**BREWS**	**Y**	**System**	**N**
**Tu**	**+**	**SE**WA	**BRE**W**S**	**Y**	**System**	**+**
**Van Durme**	**unclear**	SEWA	BR**E**WS	**N**	**N/A**	**N/A**

Workload Analyzed Using the Cumulative Complexity Model (CuCoM)

+ = transferring work to patients

- = removing work from patients

N = both transferring work to patients but providing support

Normalization Process Theory (NPT)

S = sense-making work

E = enrolling others and planning the work

W = enacting the work

A = appraising the work

Theory of Patient Capacity (BREWS)

B = biography support R = resource support

E = supportive healthcare environment

W = workload support

S = support of the social network

Outcomes Reported = Yes or No—studies that primarily focused on reporting implementation characteristics or lessons learned, and/or did qualitative analysis only are recorded as "No"

Outcome Focus = Patient-focused outcomes (e.g., quality of life, involvement in decision making, confidence in managing conditions, etc.); System-Focused Outcomes (e.g. ACIC, laboratory values, % patients meeting guideline targets, etc.); or both

Outcomes

+ = all or majority positive outcomes from intervention.

- = no studies reported completely negative outcomes.

N = mixed results; some outcomes positive, others negative or null.

### Patient work

No CCM studies acknowledged patient work or the impact of life’s work on patient health or healthcare. In six studies, the work asked of patients by the intervention was unclear, and in eight it was neutral—they asked patients to enact work, but also provided support to help patients carry out this work. Most studies (n = 19) transferred work to patients by asking them to attend more classes, more appointments, or appointments on specifically scheduled days, and by intensifying treatment. Only four studies actually took work off the patient’s plate without adding any additional work.[33–36] Examples of how to reduce patient work can be gleaned from these studies. One intervention changed the role of paramedics, such that they conducted regular home visits with patients, rather than having patients come to clinic unless absolutely warranted.[33] Another traced patients lost to follow-up by conducting home visits, and for patients unable to travel to the clinic, they introduced outreach visits.[34] Roland et al., described the evaluation of multiple pilots for care of elderly patients, which offloaded work from patients through intensive team communication about patients most at risk for admission to the hospital and rapid follow-up by phone or home visits as needed for patients.[35] In many of these pilot sites, community and social services and home-care services were deployed.[35] Finally, in a program for patients with inflammatory bowel disease, the healthcare team made a 24-hour nurse line available to all patients, so that they would not need to seek care elsewhere for urgent questions.[36] Additionally, they proactively followed-up by phone with patients who had medication changes, were on certain therapies, or who were discharged recently from the hospital, ensuring patients did not need to do the work of navigating how best and with whom to follow-up.[36]

Interventions that supported patient work most commonly supported sense-making activities or activities required to enroll others to help and to plan the work. This was accomplished through patient education, referrals to outside agencies, or with home visits. Few interventions helped patients accomplish the work or appraise whether the work was worth the effort. One way in which patients were helped to appraise their self-management actions was to set-up regular coaching calls with the patient to monitor goals and symptoms, and to change action plans as needed based on this feedback loop.[37]

### Patient capacity

Patient capacity was most often supported through the provision of resources required to carry out the work of being a patient, namely patient education materials and courses. Few implementations provided other resources or support, such as transportation or financial assistance. The next most supported element was improvement in the care environment to make it more patient-centered, typically by implementing team-based care to provide more holistic care. Very few studies supported the patients’ reframing of their biography in the face of chronic disease. Patients with chronic illness often lose the potential to fulfill important obligations and dreams in their life including the ability to care for family, work, and partake in pleasurable activities. This loss of taken-for-granted perceptions of self is called biographical disruption.[38] Furthermore, few studies supported productive interactions with the patients’ social network.

Only three studies supported all constructs of patient capacity, and these studies deserve attention as potential exemplars for future work. To highlight how supporting all elements of patients’ capacity might be accomplished consider, Smidth et al.,[39] which reported on a program for patients with COPD. They supported patient capacity through their exploration of **B**iography with illness in conversations that took an “appreciatory approach with dialogue between the patient and the health professional about the patient’s range of choices and opportunities, available treatment options and the patient’s readiness to change habits.”[39] Additionally, self-management course content supported overcoming biographical disruption through “knowledge and insight into their own psychological and physical situation, discuss and provide new inspiration for sexual life.”[39] They provided **R**esources such as a simple action card with information on exacerbations and steps to take. They improved the care **E**nvironment by encouraging a team-based approach to caring for patients with COPD, and by creating manuals for health professionals to ensure no tests were duplicated, which would have caused more work for patients. To support patients in accomplishing the **W**ork of being a patient they included regularly scheduled group self-management sessions that placed emphasis on “participatory activities with dialogue-based knowledge exchange to aid development of competences to act.”[39] Finally, they “wanted to inspire and encourage family, friends and patients to talk openly about the disease by providing disease-specific knowledge and therefore developed a webpage with information about the following issues: COPD; the support, help and aid provided by the municipality; local support groups and the general practices.”[39] This supports patients’ capacity to interact with their **S**ocial network about caring for their illness. As this exemplar demonstrates, however, even the best applications of elements of MDM tend to focus on the care of a single condition.

### Outcomes

The CuCoM postulates that if care is aligned such that patient workload and capacity are balanced, patients will be better able to access and use healthcare and enact self-care, which in-turn, should improve outcomes.[15] In line with this, we examined whether reports included any outcomes, whether the focus of those outcomes were on the patient (e.g., their confidence in managing their condition or their quality of life) or on the system (e.g. patients’ adherence to guidelines, surrogate markers, chronic care implementation efforts). Approximately two-thirds of reports included some outcome reporting. The majority of reports included system-focused outcomes only. All studies that reported outcomes reported mixed or positive results, and none had entirely negative findings. There did not appear to be a clear association between included MDM components and outcomes; however, this type of synthesis was difficult given the heterogeneity of study designs included (e.g., implementation, observational, intervention pre- post-, RCTs, etc.) and the heterogeneity of the interventions (e.g. practice facilitation vs. care manager implementation).

## Discussion

Our analysis uncovered four important findings:

1. Very few implementations of the CCM are agnostic to chronic condition type or target patients with multiple chronic conditions.
2. The primary aims of these interventions were to improve disease-specific outcomes or reduce healthcare utilization, and most were conducted in alignment with the healthcare system’s goals. Few studies focused on patient-centered outcomes, such as functional status, coping skills, or quality of life.
3. Studies primarily supported patient capacity through the provision of information resources. Few provided practical resources such as transportation or financial assistance, helped patients reframe their biography, or fostered productive interactions with their social network.
4. None of the included articles specifically mentioned patient work. Most implementations were either unclear in their impact on patient work or added to patient workload. Very few articles took work away from patients without adding new tasks.

### Implications for practice and policy

Studies evaluating the CCM reveal that they, for the most part, have not incorporated the contributions of MDM. Specific problems for complex patients with multimorbidity that could be better incorporated into CCM implementations include considering the compound effects of conditions and treatments and their interaction with the demands of life, the administrative and financial complexity of attending to multiple conditions, and the additional coordination and communication with and amongst clinicians required to care for a patient with multiple conditions.[40, 41] Incorporating the MDM construct of “treatment burden,” the impact that healthcare workload has on patient wellbeing,[22] could build on CCM implementations to better address the needs of patients with multimorbidity in whom work accumulates and often overwhelms. Treatment burden has been well documented across a number of conditions and is an important factor that can lead to nonadherence.[10, 11, 23, 24, 42, 43] Furthermore, the burden of multimorbidity, andits associated increases in treatment work, falls more often on patients of lower socioeconomic status, often times without increased clinical care or clinical funding to areas of high social deprivation.[44, 45] We saw little focus specifically on implementing the CCM specifically for patient populations of low SES, with only approximately one-third of the papers using SES as a rationale for their study or conducting their research in low-resource settings. Another one-third of papers briefly mentioned SES somewhere or adjusted for it in their analyses, and the final one-third make no mention of SES variables or considerations.

Additionally, CCM implementations could be further tailored to incorporate the MDM construct of patient capacity in order to better support patients. Patients most disrupted by their illness and care are those with limited physical, emotional, and financial capacity,[46] suggesting, at minimum, interventions should pay attention to the resources needed to support these capacities. Most implementations sought to support patient capacity through the provision of education. However, a few tried to overcome problems like financial burdens, transportation, and problematic access hours, which are well documented problems for patients with chronic conditions.[10, 11, 23, 24, 26, 43] More interventions should seek to incorporate these elements to support patient capacity.

Additionally, the implementations of the CCM in the literature did not report supporting patients as they reframed their biography with chronic illness and or supporting their interactions with their social network. While this may be a limitation in detailed reporting of intervention components, it still deserves attention. Supporting the reframing of biography is emerging as a critical component of care as it may affect many other elements of capacity such as the ability to mobilize existing resources or to gain experiential learning from successfully carrying out patient and life work.[9] Patients’ biographies include *who* the person is (e.g., a working grandmother) and *what* is most important to them (e.g., gardening and playing with grandkids). Biographical disruption is caused by *how* illness and treatment disrupt those important roles and activities (e.g., time away to attend medical appointments and pain inhibiting paid work). Chronic care can support biographical reframing by reducing the disruption caused by healthcare itself and supporting patients in conversation with health professionals and peers about changes caused by illness and strategies to cope and thrive. Of note, the American Geriatrics Society has called for at least incorporating this type of information into treatment decision making by putting forth as their first guiding principle of care for older adults with multimorbidity eliciting and incorporating patient preferences into medical decision-making.[47] However, it is also worth considering that the population of patients living with multimorbity includes patients who are not yet geriatrics patients, as well as the apparent need for supporting the patients’ biographical reframing beyond the inclusion in care decisions alone.

Finally, patients’ capacity depends in part on acting in collaboration with their social network. When the social network fails to recognize the importance of this help, understand practically what needs to be done, or is non-existent, patients struggle to mobilize capacity.[9] The Burden of Treatment Theory states: “*Interventions that maximize collective competence in enacting practical tasks, distributing help and exploiting local resources, and effect increased confidence in healthcare processes and outcomes, are therefore likely to reduce inappropriate demands on healthcare services*.[14] Three quarters of the literature examined on current implementations of the CCM did not report maximizing this collective competence, missing a critical opportunity to potentially support patient quality of life while simultaneously reducing the demands on the healthcare system. The quarter that did seek to draw on social support for patients did so by implementing group visits, promoting support groups, and tailoring education material for the social network of the patient, not just the patient individually. These strategies could be used in more CCM interventions to improve the collective competence of the patient and their social network. This recommendation is strengthened by additional reports of caregiving difficulties in caring for patients with multimorbidity, including caregivers’ frustrations with the work associated with accessing and coordinating care,[48] and higher caregiver strain for caregivers with greater numbers of caregiving tasks and lower self-efficacy.[49]

### Implications for research

The CCM has modernized healthcare to respond proactively to the common occurrence of patients with chronic illness. The model tell us what to implement (e.g., clinical information systems), but the orientation of the CCM components to better the care of complex patients with multimorbidity may benefit from the contributions of MDM. Our review demonstrates that this potential awaits evaluation. Researchers must rigorously design interventions with strong theoretical underpinnings, which are sensitive to the issues highlighted in this review. In particular, to the care patients can use to flourish through careful consideration of the complexities of care and life and the interplay of workload and capacity. Interventions with theoretical underpinnings are more likely be effective, allow replication, and to allow better identification of the components of complex interventions that actually are responsible for their effects.[25] It is important that future evaluations look at outcomes important to a variety of stakeholders, most importantly, patients, and measure not only disease-specific metrics or utilization, but also patient-centered outcomes such as treatment burden, quality of life, and functional status. The recommendation of more inclusive measures is strengthened in light of the Cochrane systematic review on interventions specifically designed for multimorbidity, where still only one-half of included study included patient-reported outcome measures.[50] In regards to other outcomes, studies that included depression as a co-morbid condition did show consistent improvements in depression-related outcomes.[50] Otherwise, the review illustrated mixed effects or no effects of interventions specifically for multimorbidity across a variety of other outcomes including clinical outcomes, healthcare utilization, medication use and adherence, and health-related patient behaviors,[50] highlighting the need to consider new approaches for this population.

### Strengths and limitations

Our findings are limited by what we could access from published reports, their protocols, and supplemental material, and in this, the provision of insufficient details about how the interventions were implemented and with what fidelity. Additionally, MDM is only one lens by which we can view multimorbidity, and to-date, whole-scale interventions that seek to implement all components of MDM within a healthcare system to reduce treatment burden and support patient capacity have not been implemented or tested. Despite these limitations, this systematic review fills important gaps in the current literature. First, while most reviews of the CCM have explored process and disease-specific outcomes,[51–55] this review critically evaluates *how* the CCM has been implemented. Furthermore, the CCM has not faced comparisons with emerging models that detail more specifically how to deliver care to patients with multimorbidity. This review accomplishes this by examining CCM implementations in light of MDM. In doing so, we have identified critical leverage points for changes in clinical practice, policy, and research that build on the substantial contributions of the CCM. Specifically, policy designers must acknowledge the cumulative work of being a patient and support critical elements of patient capacity. Based on the conceptual and theoretical underpinnings of MDM, one should expect that these changes would lead to healthcare that patients are better able to access and use, and self-care tasks that can be carried out within their existing capacity and life context.[15] Ultimately, these should translate into better patient outcomes and health system performance.

## Conclusion

As highlighted in this review, current interventions that deliver the components described in the CCM may need modifications in how they are delivered to meet the needs of the growing population with chronic multimorbidity. MDM provides a lens to consider these modifications. Specifically, interventions should be agnostic to condition type and accommodate the coexistence and interactions typical of multimorbidity. They must acknowledge patient work and its dynamic interaction with the work of everyday life. Interventions should also support patient capacity, including supporting patients’ ability to reshape their biography in chronic illness and to draw from their social networks. Implementation of interventions informed by MDM should be evaluated considering their ability to influence patient-centered outcomes, the experience of care for those receiving and those providing it, and the resource invested in their implementation.

## Supporting information

S1 Table ENTREQ checklist. Reporting standard for qualitative evidence syntheses.
(PDF)

S2 Table Methods reporting and bias protection based on TIDieR checklist criteria. Study quality appraisal.
(PPTX)

S1 Text Complete search strategy. Complete search strategy used for review.
(DOCX)
