# Supplementary material for: Does the chronic care model meet the emerging needs of people living with multimorbidity? A systematic review and thematic synthesis
Source: PLoS One. 2018 Feb 8;13(2):e0190852. doi: 10.1371/journal.pone.0190852 (PMC5805171; doi:10.1371/journal.pone.0190852)
Supplement: S2 Table — Study quality appraisal. (PPTX) [file pone.0190852.s002.pptx]

## Slide 1
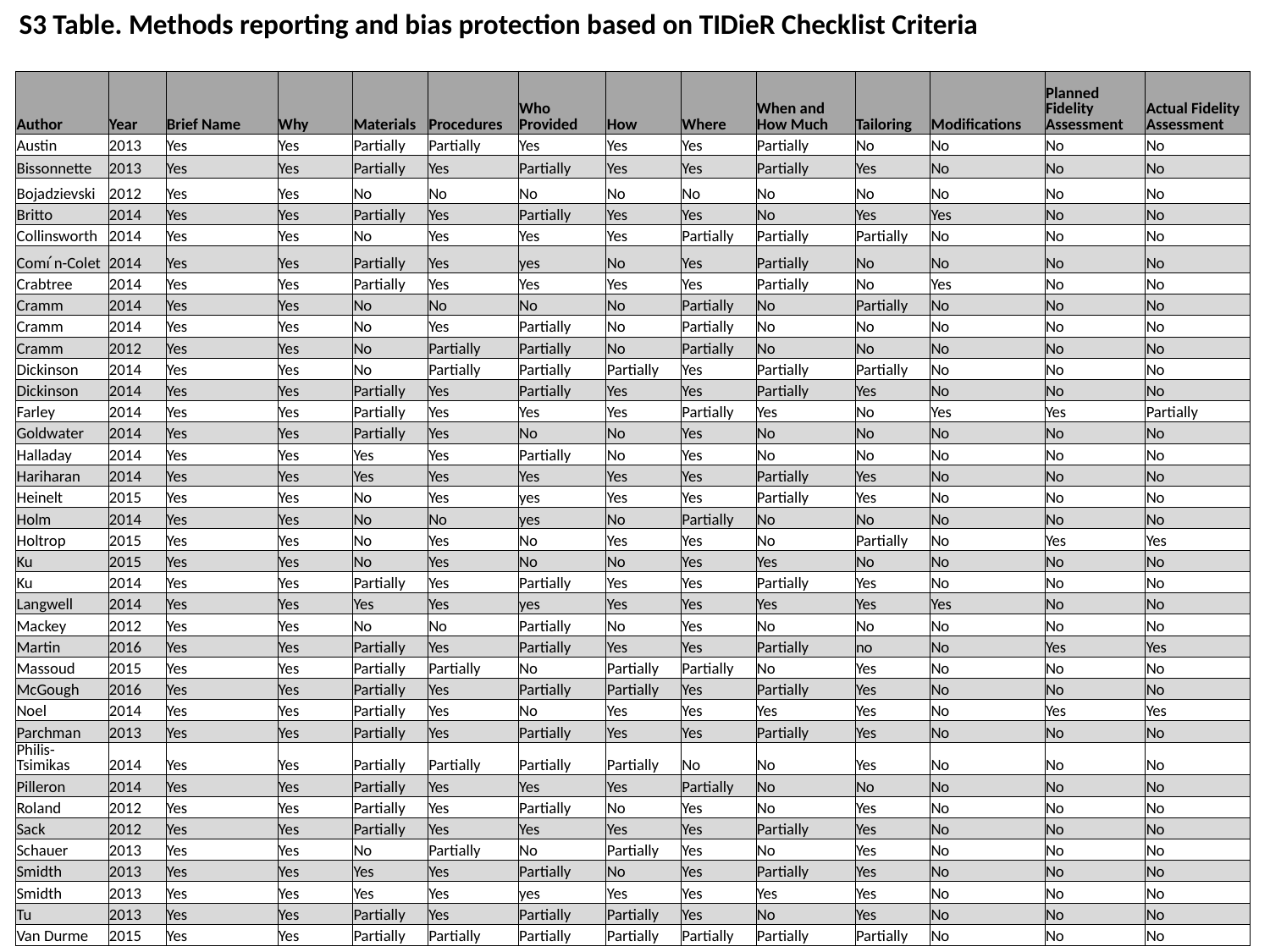

S3 Table. Methods reporting and bias protection based on TIDieR Checklist Criteria
| Author | Year | Brief Name | Why | Materials | Procedures | Who Provided | How | Where | When and How Much | Tailoring | Modifications | Planned Fidelity Assessment | Actual Fidelity Assessment |
| --- | --- | --- | --- | --- | --- | --- | --- | --- | --- | --- | --- | --- | --- |
| Austin | 2013 | Yes | Yes | Partially | Partially | Yes | Yes | Yes | Partially | No | No | No | No |
| Bissonnette | 2013 | Yes | Yes | Partially | Yes | Partially | Yes | Yes | Partially | Yes | No | No | No |
| Bojadzievski | 2012 | Yes | Yes | No | No | No | No | No | No | No | No | No | No |
| Britto | 2014 | Yes | Yes | Partially | Yes | Partially | Yes | Yes | No | Yes | Yes | No | No |
| Collinsworth | 2014 | Yes | Yes | No | Yes | Yes | Yes | Partially | Partially | Partially | No | No | No |
| Comı ́n-Colet | 2014 | Yes | Yes | Partially | Yes | yes | No | Yes | Partially | No | No | No | No |
| Crabtree | 2014 | Yes | Yes | Partially | Yes | Yes | Yes | Yes | Partially | No | Yes | No | No |
| Cramm | 2014 | Yes | Yes | No | No | No | No | Partially | No | Partially | No | No | No |
| Cramm | 2014 | Yes | Yes | No | Yes | Partially | No | Partially | No | No | No | No | No |
| Cramm | 2012 | Yes | Yes | No | Partially | Partially | No | Partially | No | No | No | No | No |
| Dickinson | 2014 | Yes | Yes | No | Partially | Partially | Partially | Yes | Partially | Partially | No | No | No |
| Dickinson | 2014 | Yes | Yes | Partially | Yes | Partially | Yes | Yes | Partially | Yes | No | No | No |
| Farley | 2014 | Yes | Yes | Partially | Yes | Yes | Yes | Partially | Yes | No | Yes | Yes | Partially |
| Goldwater | 2014 | Yes | Yes | Partially | Yes | No | No | Yes | No | No | No | No | No |
| Halladay | 2014 | Yes | Yes | Yes | Yes | Partially | No | Yes | No | No | No | No | No |
| Hariharan | 2014 | Yes | Yes | Yes | Yes | Yes | Yes | Yes | Partially | Yes | No | No | No |
| Heinelt | 2015 | Yes | Yes | No | Yes | yes | Yes | Yes | Partially | Yes | No | No | No |
| Holm | 2014 | Yes | Yes | No | No | yes | No | Partially | No | No | No | No | No |
| Holtrop | 2015 | Yes | Yes | No | Yes | No | Yes | Yes | No | Partially | No | Yes | Yes |
| Ku | 2015 | Yes | Yes | No | Yes | No | No | Yes | Yes | No | No | No | No |
| Ku | 2014 | Yes | Yes | Partially | Yes | Partially | Yes | Yes | Partially | Yes | No | No | No |
| Langwell | 2014 | Yes | Yes | Yes | Yes | yes | Yes | Yes | Yes | Yes | Yes | No | No |
| Mackey | 2012 | Yes | Yes | No | No | Partially | No | Yes | No | No | No | No | No |
| Martin | 2016 | Yes | Yes | Partially | Yes | Partially | Yes | Yes | Partially | no | No | Yes | Yes |
| Massoud | 2015 | Yes | Yes | Partially | Partially | No | Partially | Partially | No | Yes | No | No | No |
| McGough | 2016 | Yes | Yes | Partially | Yes | Partially | Partially | Yes | Partially | Yes | No | No | No |
| Noel | 2014 | Yes | Yes | Partially | Yes | No | Yes | Yes | Yes | Yes | No | Yes | Yes |
| Parchman | 2013 | Yes | Yes | Partially | Yes | Partially | Yes | Yes | Partially | Yes | No | No | No |
| Philis-Tsimikas | 2014 | Yes | Yes | Partially | Partially | Partially | Partially | No | No | Yes | No | No | No |
| Pilleron | 2014 | Yes | Yes | Partially | Yes | Yes | Yes | Partially | No | No | No | No | No |
| Roland | 2012 | Yes | Yes | Partially | Yes | Partially | No | Yes | No | Yes | No | No | No |
| Sack | 2012 | Yes | Yes | Partially | Yes | Yes | Yes | Yes | Partially | Yes | No | No | No |
| Schauer | 2013 | Yes | Yes | No | Partially | No | Partially | Yes | No | Yes | No | No | No |
| Smidth | 2013 | Yes | Yes | Yes | Yes | Partially | No | Yes | Partially | Yes | No | No | No |
| Smidth | 2013 | Yes | Yes | Yes | Yes | yes | Yes | Yes | Yes | Yes | No | No | No |
| Tu | 2013 | Yes | Yes | Partially | Yes | Partially | Partially | Yes | No | Yes | No | No | No |
| Van Durme | 2015 | Yes | Yes | Partially | Partially | Partially | Partially | Partially | Partially | Partially | No | No | No |
