# Supplementary material for: Does the chronic care model meet the emerging needs of people living with multimorbidity? A systematic review and thematic synthesis
Source: PLoS One. 2018 Feb 8;13(2):e0190852. doi: 10.1371/journal.pone.0190852 (PMC5805171; doi:10.1371/journal.pone.0190852)
Supplement: S1 Text — Complete search strategy used for review. (DOCX) [file pone.0190852.s003.docx]

**S2 Text. Complete Search Strategy.**

**Epub Ahead of Print, In-Process & Other Non-Indexed Citations, Ovid MEDLINE(R) Daily and Ovid MEDLINE(R) <1946 to Present>**

| **#** | **Searches** | **Results** | **Type** |  |  |  |
| --- | --- | --- | --- | --- | --- | --- |
|  |  |  |  |  |  |  |
| 1 | "chronic care model".mp. | 800 | Advanced |  |  |  |
| 2 | 1 and og.fs. | 301 | Advanced |  |  |  |
| 3 | 1 and implement*.mp. [mp=title, abstract, original title, name of substance word, subject heading word, keyword heading word, protocol supplementary concept word, rare disease supplementary concept word, unique identifier] | 350 | Advanced |  |  |  |
| 4 | 2 or 3 | 510 | Advanced |  |  |  |
| 5 | ..l/ 4 lg=en and yr=2012-2017 | 227 | Advanced |  |  |  |
| 6 | remove duplicates from 5 | 167 | Advanced |  |  |  |
| 7 | 5 and implement*.mp. [mp=title, abstract, original title, name of substance word, subject heading word, keyword heading word, protocol supplementary concept word, rare disease supplementary concept word, unique identifier] | 160 | Advanced |  |  |  |

Scopus:

"chronic care model"  AND  ( implementing  OR  redesign* ) )  AND  SUBJAREA ( mult  OR  agri  OR  bioc  OR  immu  OR  neur  OR  phar  OR  mult  OR  medi  OR  nurs  OR  vete  OR  dent  OR  heal  OR  mult  OR  arts  OR  busi  OR  deci  OR  econ  OR  psyc  OR  soci )  AND  PUBYEAR  >  2011  = 61
